# Supplementary figures and images for: The transcriptomic response to a short day to long day shift in leaves of the reference legume Medicago truncatula
Source: PeerJ. 2019 Mar 22;7:e6626. doi: 10.7717/peerj.6626 (PMC6432905; doi:10.7717/peerj.6626)

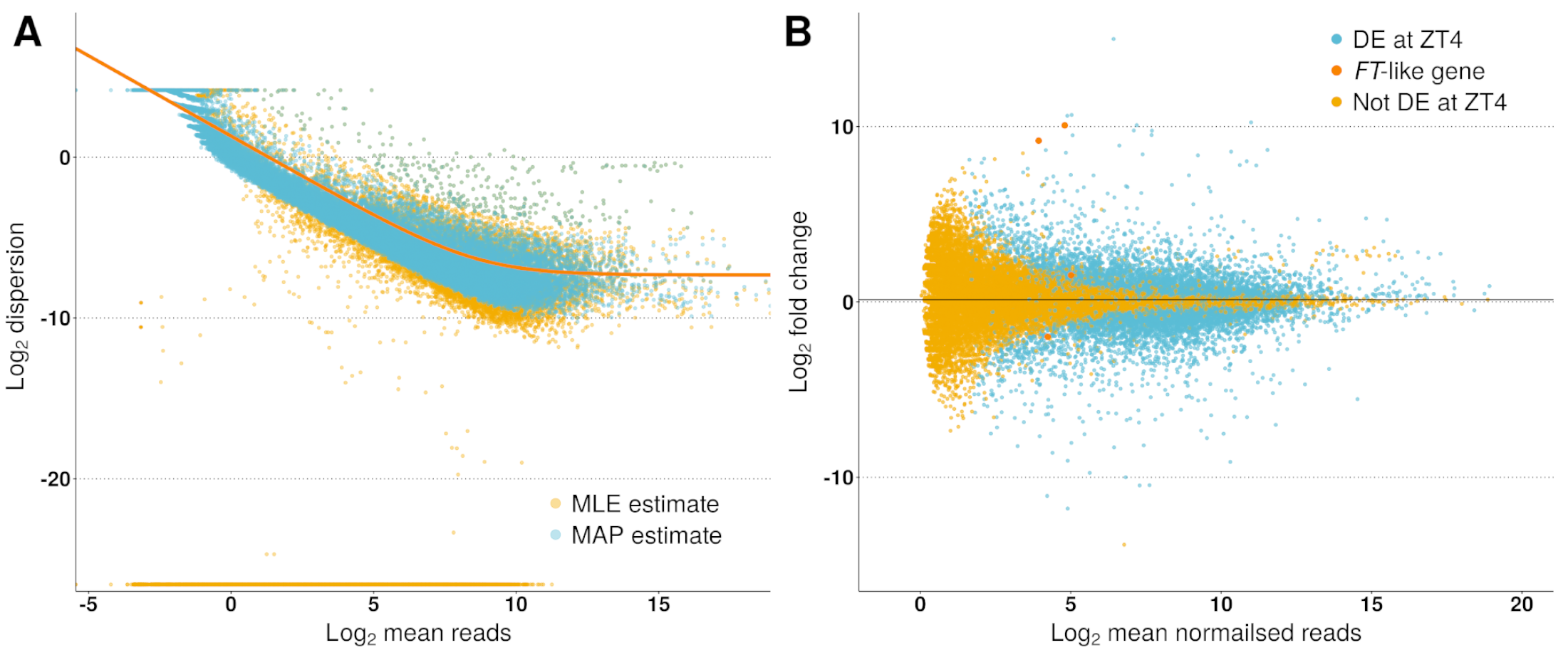

Supplement: Figure S1 — (A) is dispersion plot showing the DESeq2 model which shrinks the dispersion from the gene-wise maximum likelihood estimates (MLE; orange) to the maximum a posteriori (MAP; blue) estimates with increased log 2 mean number of reads counts per gene. (B) is a MA plot of the log 2 normailsed mean number of reads plotted against their log 2 fold-changes at ZT4. In this case the genes statistically different at ZT4 (α = 0.05) are highlighted, as are FT-like genes. [file peerj-07-6626-s001.png]

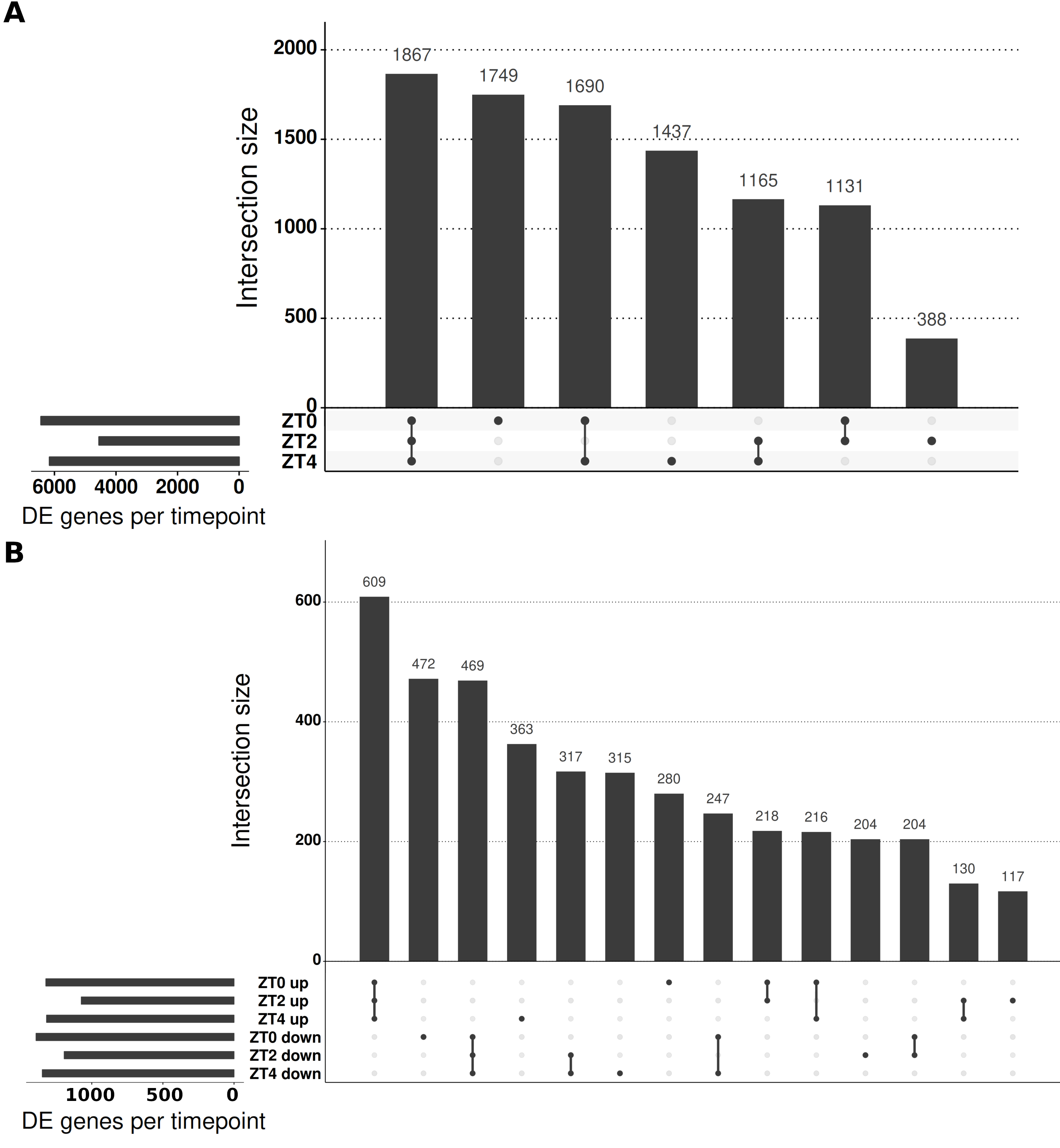

Supplement: Figure S2 — (A) contrasts the timepoints at which all 9,516 genes which alter their pattern of gene expression in response to the change of photoperiod conditions have statistically significant (α = 0.05) differences in expression. (B) contrasts the timepoints and direction of the statistically significant (α = 0.05) differences in expression in all 4,694 genes which statistically alter only the magnitude of their gene expression across the three timepoints. Membership within each group for individual genes, including non DE genes, is given in Table S8. In both subfigures the principal chart plots the size of the overlaps between timepoints and the supplementary chart presents the number of genes statistically significant (and in (B) either up or down) in LD relative to SD for each timepoint. [file peerj-07-6626-s002.png]

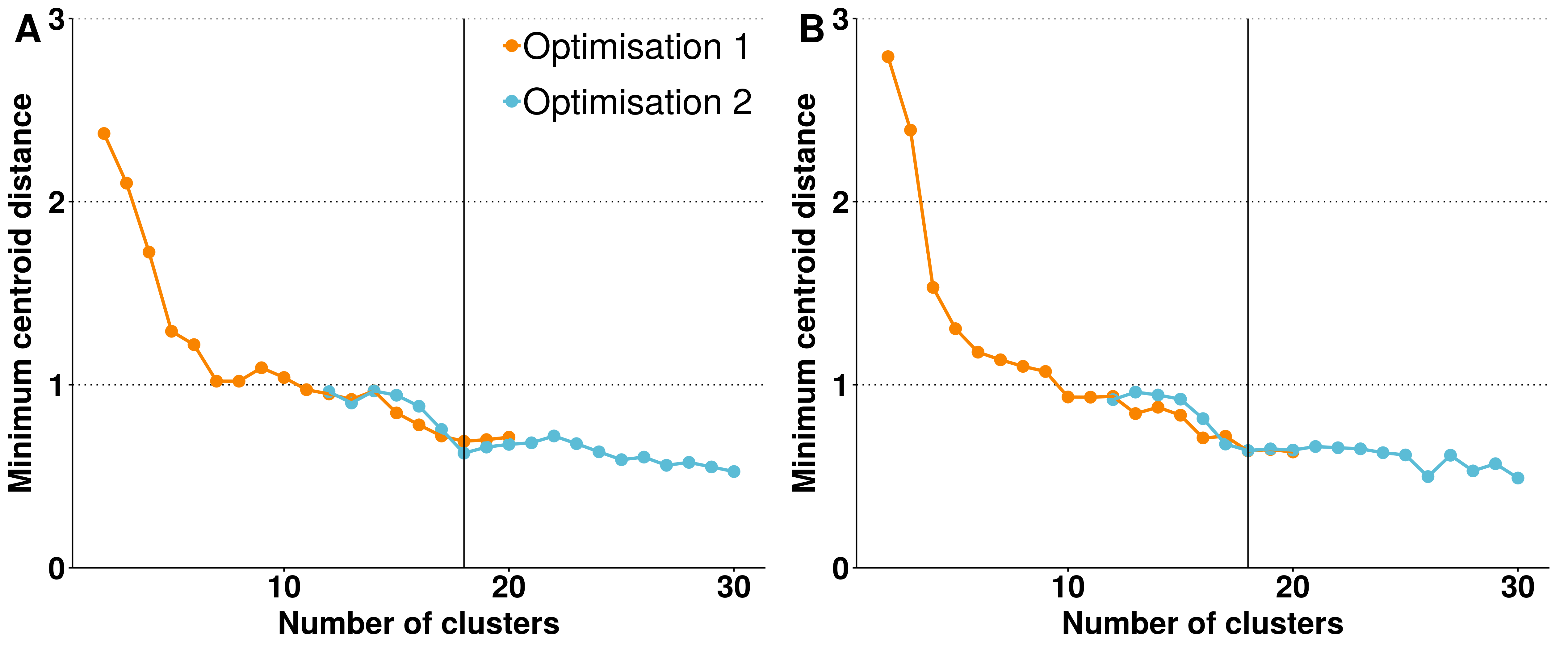

Supplement: Figure S3 — This is the result of clustering the DE genes using different values of c (number of clusters) for (A) the 9,516 genes which alter their pattern of expression and (B) the 4,694 genes differ in just the magnitude of expression. In both cases two optimisations were run (Optimisation 1: c varied between 2 and 20 in increments of 2; Optimisation 2: c varied between 12 and 30) in increments of 1. A vertical line was drawn at the point where the minimum centroid distance was judged to plateau which was taken as the optimal value of c. [file peerj-07-6626-s003.png]

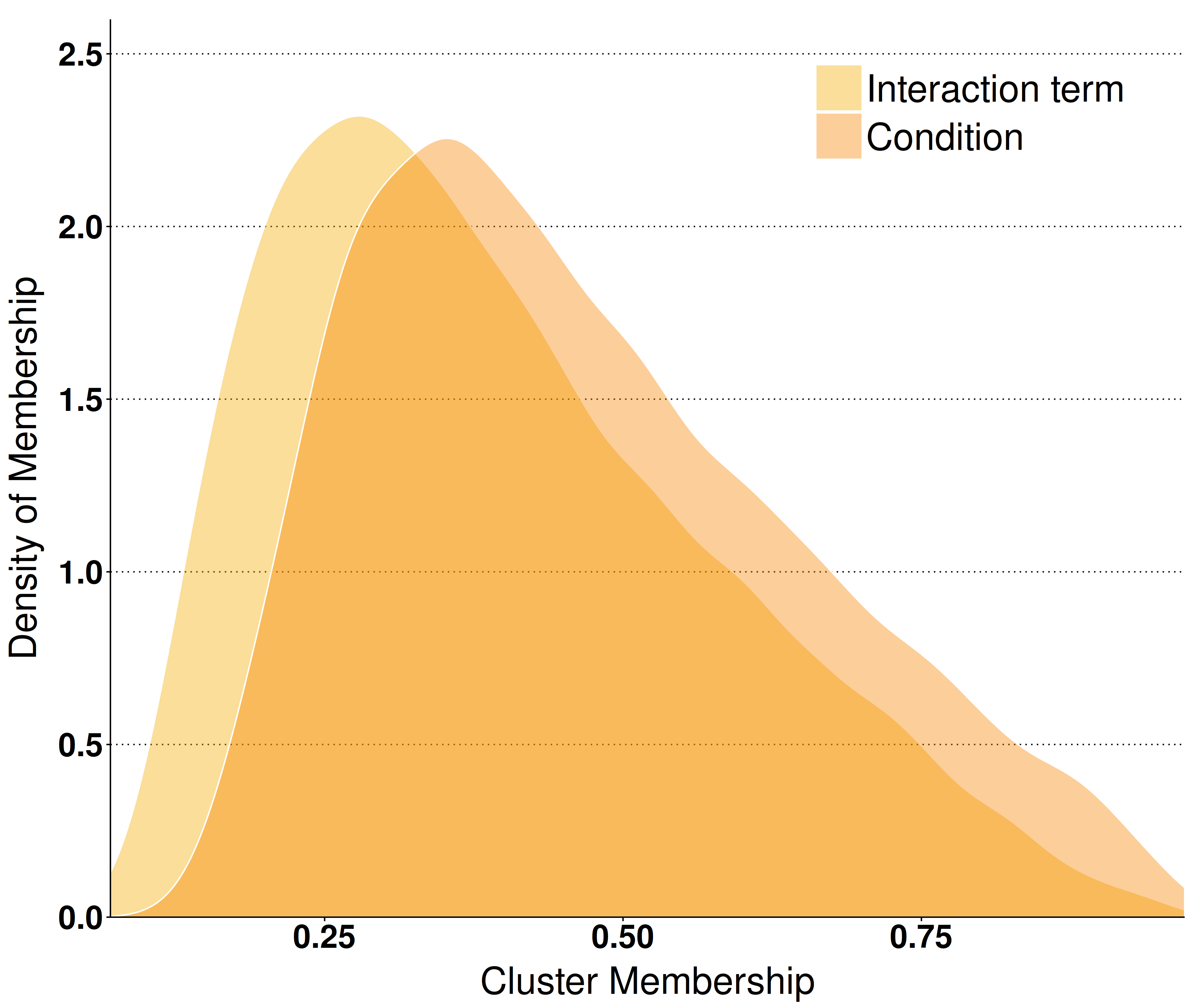

Supplement: Figure S4 — Scores range between 0 and 1 for each class of genes (9,516 for pattern of expression and 4,694 for those that differ in just magnitude) from c-means clustering. The score describes the support for an individual observation belonging to a given cluster with the cluster with the highest membership score is assigned the gene. [file peerj-07-6626-s004.png]

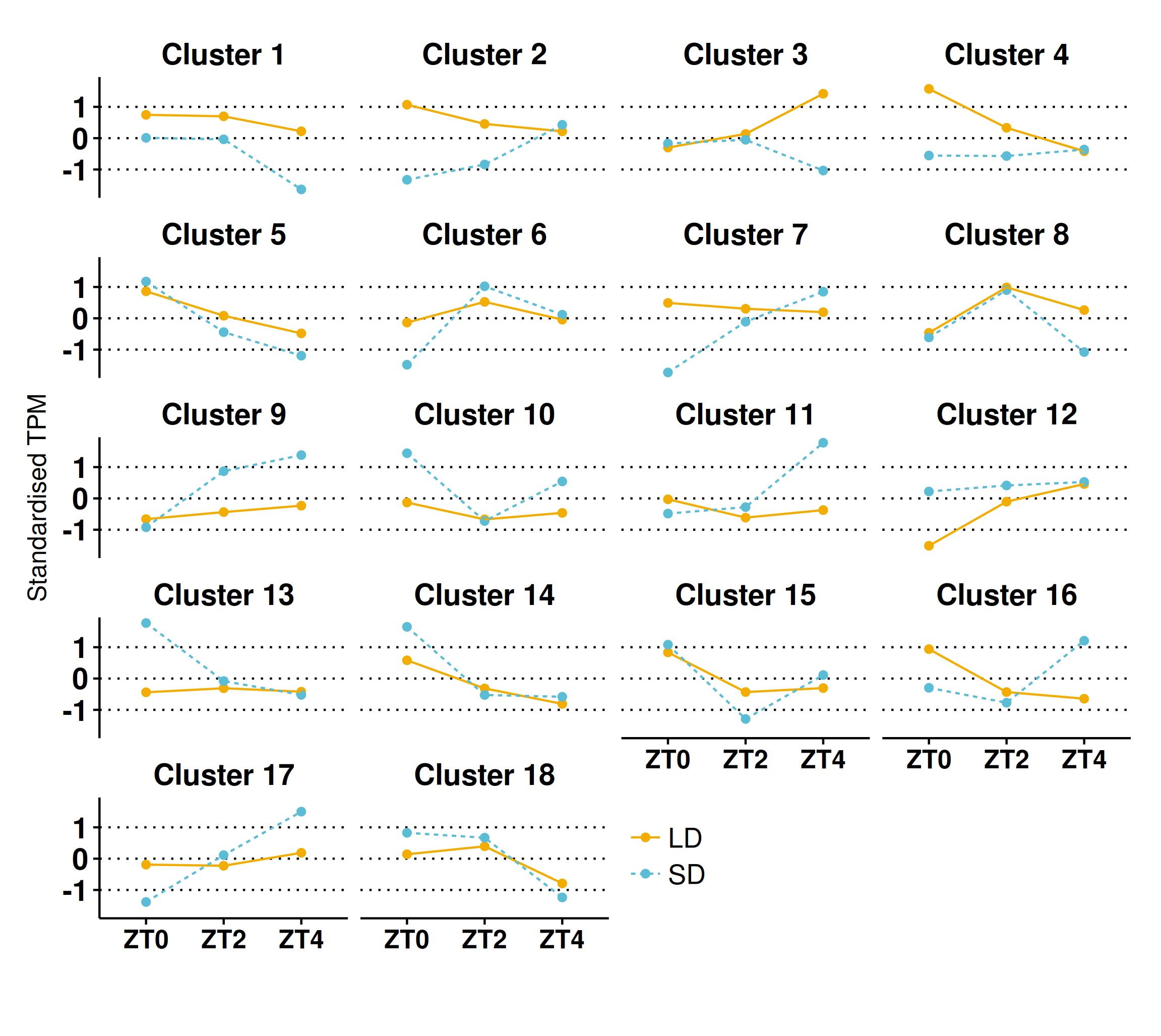

Supplement: Figure S5 — SD abundances are plotted in blue with dotted lines while LD abundances are orange with solid lines. Clustering was conducted using c-means clustering. [file peerj-07-6626-s005.png]

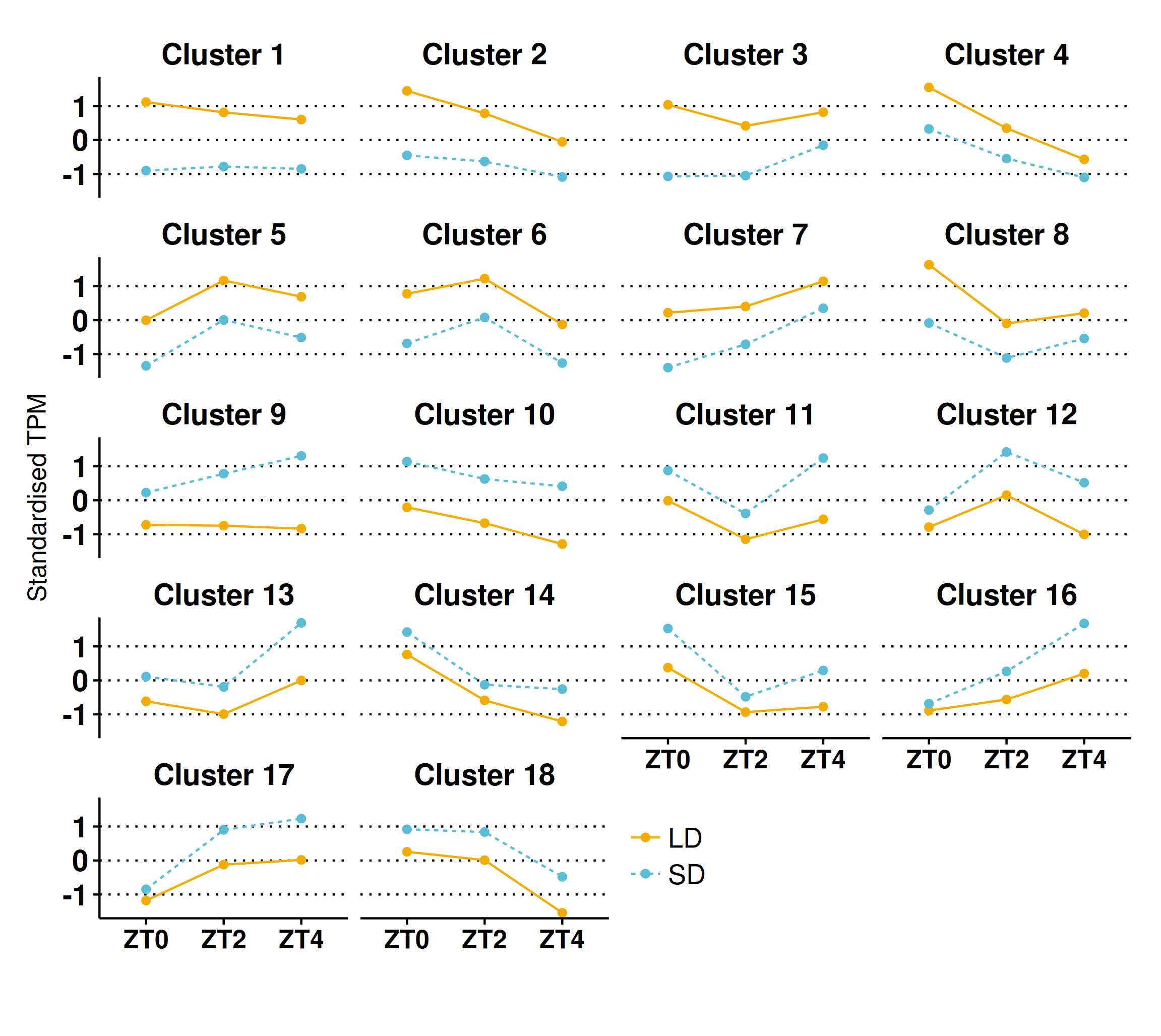

Supplement: Figure S6 — SD abundances are plotted in blue with dotted lines while LD abundances are orange with solid lines. Clustering was conducted using c-means clustering. [file peerj-07-6626-s006.png]
